# Daily Routines and Habits in Individuals with Attention Deficit Hyperactivity Disorder: A Scoping Review

**DOI:** 10.3390/bs16061000

**Published:** 2026-06-15

**Authors:** Ibrahim Almudayfir, Lama Abdulkarim, Rachael Rosenstein, Hon K. Yuen

**Affiliations:** 1 Occupational Therapy Department, College of Applied Medical Sciences, King Saud bin Abdulaziz University for Health Sciences, Riyadh 1148, Saudi Arabia; 2 Rehabilitation Science Program, University of Alabama at Birmingham, Birmingham, AL 35294, USA; 3 Independent Researcher, Riyadh 13343, Saudi Arabia; 4 Outreach Therapy, Pittsburgh, PA 15213, USA; 5 Department of Occupational Therapy, University of Alabama at Birmingham, Birmingham, AL 35294, USA

**Keywords:** ADHD, habit, routine, lifestyle, scoping review

## Abstract

This scoping review examined the current literature on routines and habits in individuals with attention deficit hyperactivity disorder (ADHD). To our knowledge, research in this area remains limited. Therefore, this review mapped which areas of daily routines are most affected in children and adults with ADHD and explored related assessments and interventions. A comprehensive search was conducted across four databases: PubMed, Scopus, CINAHL, and PsycINFO, using keywords including “attention deficit hyperactivity disorder,” “ADHD,” “routine,” “habit,” and “lifestyle.” The findings identified four main domains in which individuals with ADHD experience difficulties: sleep hygiene, feeding, physical activity, and sedentary behaviors, with sleep hygiene addressed in more than half of the included studies. Study habits were addressed in only one included study. Among the 31 included studies, six involved interventions. The review also found that no validated assessment was specifically designed to measure routines or habits in individuals with ADHD, and that broader measures of routines, habits, or lifestyle were often non-validated or developed for a single project. Overall, the existing studies were concentrated primarily in pediatric populations, with limited research involving adults. These findings highlight important gaps in the literature and underscore the need for more research on routines and habits in adults with ADHD. They also support the development of assessments and interventions that specifically address these areas.

## 1. Introduction

Attention deficit hyperactivity disorder (ADHD) is a neurodevelopmental disorder characterized by three core symptoms: inattention, hyperactivity, and impulsivity (Faraone et al., 2003). Children and adults with ADHD also experience deficits in executive functions, including response inhibition, sustained attention, working memory, and planning (Adler et al., 2017; Willcutt et al., 2005; Ziereis & Jansen, 2015). These difficulties can interfere with a person’s ability to regulate behavior, follow routines, and maintain consistent daily habits.

Routines and habits are important aspects of daily life that support functioning, health, and well-being. Consistent routines in daily activities have been associated with better functioning (Kielhofner, 2008). Although routines and habits are closely related, they are distinct concepts. A routine is generally defined as a behavior that is performed regularly and requires little conscious thought (Arlinghaus & Johnston, 2019). Routines have also been described as more organized and sequenced patterns of behavior, reflecting a broader and more structured form of action than individual habits (Clark, 2000). In contrast, habits are learned behaviors that are performed automatically and consistently, especially in familiar contexts, and they develop through repeated actions in stable environments (Taylor et al., 2023). As these behaviors are repeated over time, they become more automatic and less dependent on conscious thought or motivation (Gardner et al., 2012).

Because routines and habits are closely related, the distinction between habits and routines is not always clear. Routines are often more deliberate and structured at first, but when they are repeated in stable contexts, they may become more automatic over time. This suggests that routines and habits may be better understood not as entirely separate concepts but as related constructs that fall along a continuum of behavioral automaticity. In ADHD, difficulties may occur along this continuum because executive function challenges can interfere with the consistency, organization, and regulation needed to develop and maintain automatic patterns of daily behavior.

The Model of Human Occupation (MOHO) provides a theoretical framework to understand how routines and habits influence daily life. The model describes how daily occupations are shaped by the interaction between volition, habituation, performance skills, and the environment. MOHO positions habits and routines as components of habituation, the construct that organizes consistent patterns of daily behavior and supports participation in everyday occupations (Kielhofner, 2008). From this perspective, routines and habits can either support or limit occupational performance. When they are healthy and well-established, they can promote engagement in meaningful activities. However, when they become unhealthy, inflexible, or difficult to maintain, they may interfere with participation in desired occupations (Taylor et al., 2023).

Building on MOHO’s habituation construct, ADHD may affect occupational performance by disrupting both habits and routines (Kielhofner, 2008; Taylor et al., 2023). Because routines and habits both rely on executive functions, difficulties with attention, working memory, planning, and behavioral regulation may make it harder for individuals with ADHD to organize daily activities and repeat behaviors consistently over time (Björk et al., 2018; Willcutt et al., 2005). For example, a bedtime routine may involve stopping screen use, preparing for the next day, brushing teeth, and going to bed at a consistent time. When planning, attention shifting, or behavioral regulation are disrupted, this routine may become inconsistent, and the specific habits within it may be less likely to become automatic. Over time, this disruption in habituation may affect sleep quality, next-day energy, school or work performance, and participation in meaningful occupations. Consistent with this explanation, the findings from previous studies showed that individuals with ADHD experience difficulties maintaining healthy routines and habits across several lifestyle domains.

Research indicates that, across the lifespan, individuals with ADHD frequently develop unhealthy routines and habits, including unhealthy dietary patterns (Howard et al., 2011), sleep habits (Sciberras et al., 2017), and lifestyle habits, such as limited physical activity levels and increased screen time (van Egmond-Fröhlich et al., 2012). These unhealthy routines can negatively affect daily activities and overall health (van Egmond-Fröhlich et al., 2012). For example, unhealthy dietary habits have been linked to obesity among individuals with ADHD (Azadbakht & Esmaillzadeh, 2012; van Egmond-Fröhlich et al., 2012), and poor sleep habits correlate with more fragmented sleep, shorter sleep duration, and overall poorer sleep quality (Sciberras et al., 2017). Moreover, children with ADHD often spend excessive time using screens (Islam et al., 2024), a tendency that may contribute to the increased social and behavioral difficulties (Panjeti-Madan & Ranganathan, 2023).

Previous research on ADHD has primarily examined specific outcomes, such as sleep, physical activity, and sedentary behaviors (Advokat et al., 2011; Arias-Mera et al., 2023; Ayraler Taner et al., 2022; Bustamante et al., 2016). However, to our knowledge, no previous review has systematically mapped the literature to identify which areas of daily routines are most affected in children and adults with ADHD. In addition, little is known about how routines and habits are currently measured in ADHD research. Therefore, this scoping review aims to map the existing literature and explore how routines and habits are currently measured in this field.

## 2. Methods

This scoping review followed the methodological framework proposed by Arksey and O’Malley (2005), which consists of five key stages: (1) identifying the research question; (2) conducting a comprehensive search to locate relevant studies; (3) selecting studies that meet inclusion criteria and align with the review’s objectives; (4) extracting and organizing data to map the key findings; and (5) summarizing, analyzing, and presenting the results in a narrative format to describe the current state of the literature.

### 2.1. Identifying the Research Question

This scoping review aimed to examine how routines and habits among individuals with ADHD are addressed in the literature. It focused on identifying which areas of daily activities are most affected and what assessments and interventions researchers have used to examine them. To provide a broad overview of the literature, a range of study designs, including observational and intervention studies, were included. The population of interest was children and adults with ADHD. The primary outcomes of interest were routine- and habit-related difficulties across daily activities, as well as the assessments and interventions used in this area. The research question guiding this review was: What is known about routine- and habit-related difficulties in individuals with ADHD, and what assessments and interventions have been used to address these areas?

### 2.2. Locating Relevant Studies

A comprehensive literature search was conducted to identify relevant studies published before February 2025 across four major databases: PubMed, Scopus, CINAHL, and PsycINFO. The search strategy was developed based on the key components of the research question, incorporating terms related to ADHD, routines, habits, and lifestyle. The search was carried out by a librarian with expertise in systematic and scoping reviews. Boolean operators were used to structure the search; “OR” was applied to combine the synonyms within each search component, including population, intervention, comparator, and outcome (PICO) components, while “AND” was used to link the PICO components. Because researchers have defined and characterized routines and habits in diverse ways, no restrictions were placed on specific domains, and all relevant aspects of routines and habits were considered. A total of 5890 studies were identified and screened for eligibility. All studies were imported into Covidence for title/abstract and full-text screening. The full search strategy for each database is provided in Supplementary Materials.

### 2.3. Selecting Appropriate Studies

To be included in the review, studies had to meet the following criteria: (1) involved child or adult participants diagnosed with ADHD; (2) examined routines or habits as either an intervention component or an outcome of interest; and (3) were published in peer-reviewed journals in English. Studies were excluded if they: (1) focused exclusively on the effects of ADHD medication on routines or habits; (2) primarily investigated health-risk behaviors that are not common to pre-adolescents, such as smoking, alcohol consumption, substance abuse, unprotected sexual activity, and driving while distracted; or (3) used only qualitative methods. Qualitative studies were excluded because the aim of this scoping review was not only to map the areas of routines and habits examined in ADHD research, but also to identify how these constructs were measured and evaluated across studies. Restricting the review to quantitative studies allowed for a more consistent synthesis of outcomes, intervention components, and measurement approaches aligned with the review objectives.

Based on the Preferred Reporting Items for Systematic Reviews and Meta-Analyses (PRISMA) guidelines, two reviewers (I.A., L.A.) independently screened all titles and abstracts for eligibility using Covidence software. Full-text articles were then independently reviewed by two reviewers (I.A., R.R.). Any disagreements were resolved through discussion; when consensus could not be reached, they were resolved by a third independent reviewer (H.K.Y.). Inter-rater reliability statistics (e.g., Cohen’s kappa) were not calculated prospectively; instead, screening agreement was managed through the discussion-and-third-reviewer process.

Formal quality appraisal of the included studies was not conducted because the purpose of this scoping review was to map the existing evidence on routines and habits in individuals with ADHD. This decision is further addressed in the Limitations section.

### 2.4. Extracting and Organizing Data

We charted data from the included studies using Excel spreadsheets designed specifically for this review. The following data were extracted from each study: first author, publication year, country, study design, participant characteristics, outcome measures, and main findings. For the intervention studies, additional data were extracted on intervention characteristics, including intervention content, format, duration, and targeted outcomes. To ensure the accuracy of the extracted data, the spreadsheet was reviewed and revised by the second reviewer (L.A.).

### 2.5. Summarizing, Analyzing, and Reporting the Results

The reporting began with a basic numerical summary of the study characteristics, followed by a thematic analysis developed from these studies. We outlined key similarities and differences reported across the included studies. We then used the charted information to guide the analysis and support the presentation of findings. This approach allowed us to use a consistent method of reporting and to organize the extracted data in a table according to the identified themes, which facilitated drawing conclusions from the included studies (Arksey & O’Malley, 2005).

## 3. Results

### 3.1. Study Characteristics

**Figure 1:**
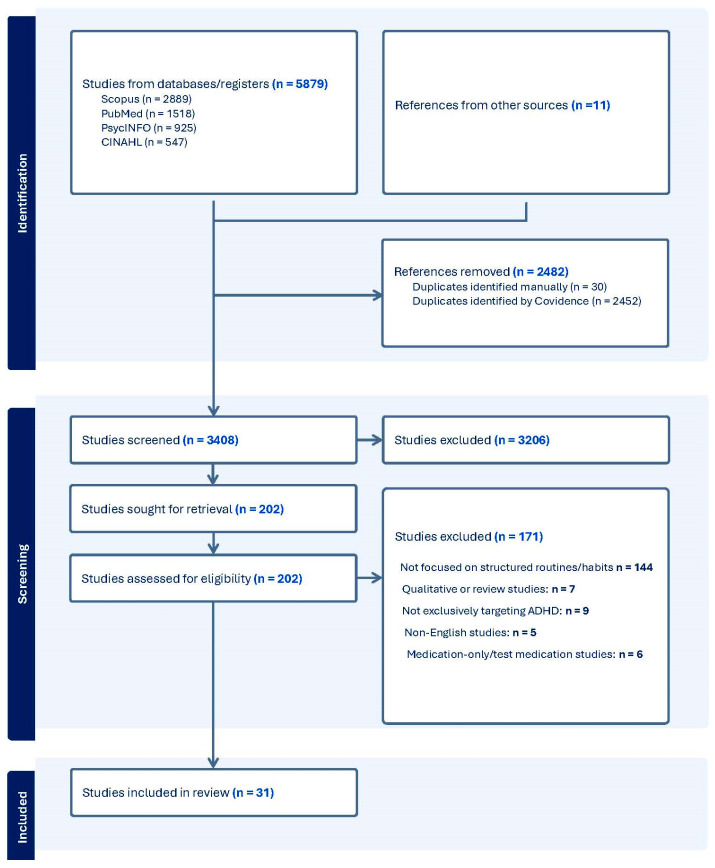
PRISMA flow diagram.

The initial database search identified 5879 studies. In addition, 11 studies related to sleep hygiene were identified through a previous systematic review (Nikles et al., 2020), which brought the total number of studies to 5890. Of these, 2452 duplicate records were automatically removed using Covidence, and an additional 30 duplicates were manually excluded. During title and abstract screening, 3206 studies were excluded, leaving 202 studies for full-text review, which were assessed against eligibility criteria. A total of 171 studies that did not meet eligibility criteria were excluded, and 31 studies were included in the final review (Figure 1).

Among the included studies, six were intervention studies, and 25 were exploratory studies. The intervention studies included one pilot feasibility and acceptability trial (Ola et al., 2021), three randomized controlled trials (Corkum et al., 2016; Hiscock et al., 2015; Shokravi et al., 2016), and two pre–post design studies (Peppers et al., 2016; Sonne et al., 2016). These studies were conducted in the United States (Ola et al., 2021; Peppers et al., 2016), the United Kingdom (Sonne et al., 2016), Iran (Shokravi et al., 2016), Australia (Hiscock et al., 2015), and Canada (Corkum et al., 2016), and all focused on sleep hygiene or sleep-related habits (Table 1). The exploratory studies were conducted across more than 10 countries and included a range of study designs, most commonly cross-sectional studies (Table 2).

The evidence base focused primarily on pediatric populations and the sleep domain. Of the 31 included studies, 27 focused on children, two focused on adults, and two included mixed age groups, ranging from adolescents to adults. The included studies also used different measures to assess routines and habits, including both validated and non-validated tools (Table 3). 

### 3.2. Thematic Analysis

Four domains were identified across the included studies: sleep hygiene, feeding, physical activity, and sedentary behaviors. However, the evidence was unevenly distributed across these domains. Sleep hygiene accounted for most of the literature and was the only domain supported by intervention studies (Corkum et al., 2016; Hiscock et al., 2015; Ola et al., 2021; Peppers et al., 2016; Shokravi et al., 2016; Sonne et al., 2016). In contrast, feeding, physical activity, and sedentary behaviors were examined mainly through exploratory studies.

#### 3.2.1. Sleep Hygiene

Sleep is one of the most studied areas in ADHD research. Individuals with ADHD often experience irregular sleep patterns and routines, which are associated with poor sleep quality (Hong et al., 2021). These patterns include delayed sleep onset, difficulty falling asleep, irregular bedtime routines, increased night waking, greater daytime sleepiness, and bedtime resistance (Becker et al., 2016; Eyuboglu & Eyuboglu, 2018; Holton & Nigg, 2020; Islam et al., 2024; Noble et al., 2011; Rodopman-Arman et al., 2011; Sciberras et al., 2017; Wannapaschaiyong et al., 2024).

Poor sleep quality was also consistently reported among children with ADHD when compared with typically developing peers (Eyuboglu & Eyuboglu, 2018; Hong et al., 2021; Rodopman-Arman et al., 2011). Several studies further suggested that irregular sleep patterns were associated with greater ADHD symptom severity (Eyuboglu & Eyuboglu, 2018; Holton & Nigg, 2020; Islam et al., 2024), behavioral difficulties (Wannapaschaiyong et al., 2024), and other sleep-related difficulties, including shorter sleep duration (Gomes et al., 2023; Sciberras et al., 2017) and difficulty waking in the morning (Becker et al., 2016). In contrast, consistent bedtime routines, such as having a set bedtime, were associated with better sleep and behavioral outcomes, including shorter sleep onset latency and improved emotional and behavioral regulation (Golos et al., 2024).

Most studies in this domain used the Children’s Sleep Habits Questionnaire (CSHQ) as the primary outcome measure (Becker et al., 2016; Eyuboglu & Eyuboglu, 2018; Gomes et al., 2023; Hong et al., 2021; Nikles et al., 2020; Noble et al., 2011; Ola et al., 2021; Rodopman-Arman et al., 2011; Sciberras et al., 2017; Shokravi et al., 2016; Sonne et al., 2016; Wannapaschaiyong et al., 2024). One study used the Family Routines Inventory to assess overall family routines (Golos et al., 2024), while two studies relied on non-validated questionnaires (Holton & Nigg, 2020; Islam et al., 2024).

Sleep routines and habits are a major concern for individuals with ADHD, yet few interventions target this area. Of the 31 studies in this review, only six involved interventions, which mainly focused on sleep (Table 1). Across these studies, interventions generally improved bedtime resistance, sleep onset latency, sleep duration, daytime sleepiness, and overall sleep disturbance, and two reported reductions in ADHD symptoms (Hiscock et al., 2015; Sonne et al., 2016). One feasibility study found that families rated consistent sleep routines as one of the most helpful lifestyle components (Ola et al., 2021). Sleep was the most extensively studied domain in this review, and the only one supported by intervention evidence; the remaining domains rely primarily on exploratory research.

#### 3.2.2. Feeding

Feeding was the second most studied domain in this review. All studies in this domain were exploratory and focused on pediatric samples. Findings across these studies showed that children with ADHD commonly experienced unhealthy diets (Halt et al., 2023; E. J. Kim et al., 2014; K. M. Kim et al., 2018; Rojo-Marticella et al., 2022; San Mauro Martin et al., 2021; Wu et al., 2016), irregular and disorganized mealtime routines (Bussing et al., 2016; Halt et al., 2023; Tong et al., 2016; Zhang et al., 2024), and overeating behaviors (Halt et al., 2023; E. J. Kim et al., 2014). These irregular feeding habits may contribute to nutritional difficulties in children with ADHD, including lower vegetable intake and higher consumption of fast food and sweets (Halt et al., 2023; K. M. Kim et al., 2018; Rojo-Marticella et al., 2022). These irregular feeding habits may also be associated with behavioral challenges, such as skipping breakfast on weekdays (Halt et al., 2023) and using electronic devices during mealtimes (Tong et al., 2016). In addition, two studies linked ADHD-related symptoms, particularly impulsivity, with unhealthy dietary habits (K. M. Kim et al., 2018; Wu et al., 2016). One study further reported that adherence to healthy feeding recommendations was associated with fewer ADHD-related clinic visits (Loewen et al., 2020). Overeating is also common among individuals with ADHD (Halt et al., 2023; E. J. Kim et al., 2014). One large study found that children with the disorder were more likely to overeat compared to typically developing peers, and this pattern was associated with higher body mass index (E. J. Kim et al., 2014).

Two studies used the Food Frequency Questionnaire to measure children’s dietary intake (Loewen et al., 2020; Rojo-Marticella et al., 2022), and one used the Mediterranean Diet Quality Index for Children and Adolescents (KIDMED) test to assess children’s adherence to the diet (San Mauro Martin et al., 2021). The remaining studies used self-reported or non-validated questionnaires to assess feeding habits.

#### 3.2.3. Physical Activity

Most studies of physical activity were exploratory, and evidence was largely drawn from samples of children, although similar patterns were also reported in adolescents and adults. Individuals with ADHD were less likely to engage in structured or vigorous physical activities, such as sports (García-Hermoso et al., 2024; Halt et al., 2023; Holton & Nigg, 2020; Wu et al., 2016) or regular exercise (Islam et al., 2024). They often had an inactive lifestyle, which can negatively affect their daily functioning and lead to unintended consequences (Björk et al., 2018; Halt et al., 2023; Holton & Nigg, 2020; Islam et al., 2024), including increased ADHD symptoms (Holton & Nigg, 2020), sleep difficulties (Hong et al., 2021), and obesity (García-Hermoso et al., 2024).

Individuals with ADHD also had difficulty adhering to regular physical activity routines (Wu et al., 2016). Despite these challenges, research indicated that structured and consistent physical activity routines were associated with positive health outcomes in children with ADHD. One study showed that children who participated in structured physical activity with a coach one to three times per week had fewer clinic visits related to ADHD (Wu et al., 2016). Another found that children who followed healthier lifestyle routines, including regular physical activity, had significantly fewer such visits (Loewen et al., 2020).

Measures of physical activity varied across studies and often relied on non-validated questionnaires (Björk et al., 2018; Halt et al., 2023; Holton & Nigg, 2020; Islam et al., 2024; Wu et al., 2016), although some studies used more established tools such as the Children’s Leisure Activities Study Survey–Parent Questionnaire (CLASS; Hong et al., 2021) and the Physical Activity Questionnaire for Children (PAQ-C; Loewen et al., 2020).

#### 3.2.4. Sedentary Behaviors

Sedentary behaviors are defined as activities that require very low energy and are typically done while sitting (Suchert et al., 2017) and can be categorized as screen-based and non-screen-based. These behaviors were commonly reported in both adults with ADHD (Björk et al., 2018; Islam et al., 2024) and children with ADHD (Golos et al., 2024; Holton & Nigg, 2020; San Mauro Martin et al., 2021; Tong et al., 2016; Wu et al., 2016; Zhang et al., 2024), although most studies in this domain were based on pediatric samples. Studies further suggested that individuals with ADHD may be more sedentary than the general population. Björk et al. (2018) observed that adults with ADHD spent more time in sedentary activities compared to adults without ADHD. Several studies showed that children with ADHD tended to spend more time than peers without ADHD on screen-based sedentary behaviors (Golos et al., 2024; Holton & Nigg, 2020; Suchert et al., 2017). Excessive screen time was linked to serious consequences, including increased ADHD symptoms (Islam et al., 2024; Suchert et al., 2017) and sleep difficulties (Ceranoglu, 2018; Hong et al., 2021). Islam et al. (2024), for example, found a strong correlation between ADHD symptoms in adolescents and young adults and problematic smartphone use, defined as an inability to regulate engagement with the device. Moreover, other studies found that children with ADHD spent more than 3 h daily using screens (San Mauro Martin et al., 2021; Zhang et al., 2024). In addition, Tong et al. (2016) noted that children with ADHD often use screens while doing other activities, such as eating or preparing for sleep, which may disrupt routines and increase obesity risk. In contrast, less evidence linked ADHD symptoms to sedentary activities that do not involve screens, such as reading or doing homework (Suchert et al., 2017).

Among the included studies, validated tools used to measure sedentary behavior included the Young Children’s Participation and Environment Measure (YC-PEM), in which screen time was included as part of home-based sitting activities (Golos et al., 2024); the short version of the Smartphone Addiction Scale (SAS-SV), which assesses screen use (Islam et al., 2024); and an adaptive survey developed by Zabinski et al. (2007) to measure sedentary behaviors (Suchert et al., 2017). The other studies used non-validated questionnaires to assess sedentary behaviors (Björk et al., 2018; Holton & Nigg, 2020; Hong et al., 2021; San Mauro Martin et al., 2021; Tong et al., 2016; Wu et al., 2016; Zhang et al., 2024).

## 4. Discussion

### 4.1. Common Areas of Routine and Habit Difficulties in ADHD

The review identified several common areas in which individuals with ADHD experience difficulties related to routines and habits. The findings showed that these challenges were most frequently reported in four domains: sleep hygiene, feeding, physical activity, and sedentary behaviors. However, not all included studies reported significant disruptions in routines or habits among individuals with ADHD, suggesting that these difficulties may vary across domains, study populations, and developmental stages. Sleep hygiene was the most frequently examined domain and the only area in which intervention studies were identified, suggesting that sleep-related routines have received the greatest research attention within the current literature. In contrast, feeding, physical activity, and sedentary behaviors were supported mainly by exploratory studies, indicating that intervention research in these areas remains underdeveloped.

Study habits were addressed in only one included study, which focused on university students with ADHD (Lindstrom et al., 2020). The findings showed that students with ADHD reported feeling less prepared for exams, experiencing greater test anxiety, and demonstrating lower academic performance, despite reporting similar study time, attendance, planning, and study environments compared with peers without ADHD.

From a MOHO perspective, the clustering of findings across sleep, feeding, physical activity, and sedentary behavior suggests that ADHD-related difficulties may reflect broader disruptions in habituation rather than isolated behavior problems. These domains all require repeated behaviors, sequencing, and consistency across daily contexts, which may be difficult for individuals with ADHD to maintain.

The findings of this review also raise an important question about the direction of the relationship between ADHD symptoms and disrupted routines. Cross-sectional studies can identify associations between ADHD and lifestyle-related behaviors, but they cannot determine whether routine disruptions contribute to ADHD-related difficulties, whether ADHD symptoms make it harder to maintain healthy routines, or whether both processes occur together. For example, Hong et al. (2021) suggested that screen time, diet, and physical activity may mediate the relationship between ADHD and sleep difficulties. However, because of the cross-sectional design, the direction of these relationships cannot be established. The two longitudinal studies included in this review provide stronger evidence for understanding how routines and ADHD-related outcomes may develop over time. Wu et al. (2016) followed children from age 10 to 18 and found that unhealthy routines were associated with greater use of ADHD-related services, while Loewen et al. (2020) followed children from age 10 to 14 and found that greater adherence to health recommendations related to diet and physical activity was associated with fewer ADHD-related physician visits. Although these studies help show that routines and ADHD-related outcomes are connected across development, they still do not fully explain the mechanisms or direction of this relationship. Therefore, developmental trajectories remain an important area for future research, particularly through longitudinal studies that directly examine whether routine disruptions predict later ADHD symptoms, whether ADHD symptoms predict later routine difficulties, or whether these relationships are bidirectional.

### 4.2. Assessment of Routines and Habits in ADHD

An important finding of this review was the variation in how routines and habits were assessed across studies. Sleep was the most consistently measured domain, with many studies using the Questionnaire (CSHQ; Becker et al., 2016; Eyuboglu & Eyuboglu, 2018; Gomes et al., 2023; Hong et al., 2021; Noble et al., 2011; Sciberras et al., 2017; Shokravi et al., 2016; Sonne et al., 2016). Although the CSHQ is often used to assess sleep-related difficulties, it was designed to assess both sleep habits and sleep disturbances rather than sleep quality alone. Several of its domains are behaviorally based and reflect repeated bedtime or sleep patterns, while other domains focus more on sleep disturbance or sleep quality. This likely contributed to the stronger and more coherent evidence base in the sleep literature. In contrast, feeding, physical activity, and sedentary behaviors were more often assessed using self-report, non-validated, or study-specific questionnaires, with only a small number of studies using validated tools.

None of the included studies used a validated assessment specifically designed to measure the overall routines or habits in individuals with ADHD. In addition, tools used to assess broader constructs, such as overall routines, habits, or lifestyle patterns, were typically non-validated measures or questionnaires developed for a single project. This limits consistency in measurement across studies and makes it difficult to compare findings or draw firm conclusions about routines and habits in this population.

### 4.3. Factors That Influence Routines and Habits in ADHD

The reviewed studies suggest that routines and habits in ADHD are shaped not only by individual behaviors but also by environmental and contextual factors. This was especially clear in the sleep studies. The three sleep hygiene interventions that were focused on parent education were associated with improvements in children’s sleep-related outcomes (Hiscock et al., 2015; Peppers et al., 2016; Shokravi et al., 2016). In addition, other studies highlighted the role of the family environment more broadly. For example, Zhang et al. (2024) found that family relationships and family lifestyle were significantly associated with ADHD, and Golos et al. (2024) showed that family routines were significantly associated with home participation and quality of life. These findings suggest that family structure and home routines may serve as important supports for routine development, particularly in childhood.

Age is another important factor. The included studies were heavily concentrated in pediatric samples, with only a small number of adult or mixed-age studies. This is important because routines and habits may not function in the same way across the lifespan. In childhood, routines are often externally structured by parents, schools, and caregivers. In adulthood, they may depend more on self-management, planning, and independent regulation.

Cultural context is another factor that may influence the development and maintenance of healthy routines and habits. Because the included studies were conducted in countries with different social expectations and daily norms, what is considered a healthy or regular routine may vary across settings. In some cultures, healthy routines are introduced and reinforced from an early age, whereas in others they may receive less emphasis.

### 4.4. Limited Attention to Habits and Routines in ADHD Rehabilitation

Habits and routines have received limited attention in ADHD rehabilitation, despite their important role in supporting activity performance and participation in daily life. The findings of this review showed that sleep hygiene, feeding, physical activity, and sedentary behaviors were often interrelated rather than isolated areas of difficulty. Disruptions in one area appeared to affect other parts of daily life, including academic performance, health-related outcomes, emotional regulation, and overall participation. From a MOHO perspective, this pattern suggests that the difficulties identified in the literature reflect broader disruptions in habituation among individuals with ADHD. These disruptions may be related to executive function deficits, which can make it difficult to organize, repeat, and maintain daily behaviors over time. The findings also showed that disruptions in habits and routines can affect other daily activities. For example, poor sleep routines may affect next-day attention and academic performance, while high sedentary behavior and irregular physical activity may influence health, energy, and engagement in meaningful activities. However, most studies examined these behaviors separately, with limited attention to how they interact within larger routines across home, school, work, and community contexts. Applying MOHO to these findings highlights the need for rehabilitation research and interventions that address habits and routines as interconnected patterns that support occupational performance and participation, rather than focusing only on isolated behaviors.

This limited attention was also reflected in the intervention literature. From the included studies, only one intervention (LEAP) specifically focused on promoting healthy routines and habits in children with ADHD (Ola et al., 2021). The other five intervention studies mainly targeted sleep hygiene and sleep-related habits (Corkum et al., 2016; Hiscock et al., 2015; Peppers et al., 2016; Shokravi et al., 2016; Sonne et al., 2016). This suggests that, although difficulties in routines and habits have been identified across several areas of daily life, intervention efforts remain limited in scope and are mostly concentrated on sleep.

### 4.5. Future Research

Future research should give greater attention to routines and habits in ADHD. Although sleep-related routines have been the most extensively studied, other areas remain underexamined. There is a clear need for a validated tool that specifically assesses routines and habits in individuals with ADHD across key daily domains, such as sleep, feeding, physical activity, sedentary behavior, self-care, school, and work. Future longitudinal studies should also examine routines as broader patterns of daily life rather than as isolated behaviors, with repeated assessments over time to better understand how routines and ADHD-related difficulties influence each other across development. More research involving adults is also needed, particularly young adults transitioning to college, employment, and independent living, to better understand how routines and habits affect daily functioning across the lifespan.

### 4.6. Limitations

This review has several limitations that should be considered when interpreting the findings. Most of the included studies used cross-sectional designs, which limits the ability of the existing literature to clarify the direction of the relationship between ADHD and difficulties in routines and habits. Although establishing causality was not an objective of this scoping review, the strong reliance on cross-sectional evidence reduces the confidence with which these associations can be interpreted.

The included studies largely focused on pediatric populations. This is important because routines and habits may vary across developmental stages, and the factors that support or disrupt their development and maintenance in adulthood may differ from those observed in childhood. As a result, the findings may not fully reflect the experiences of adults with ADHD.

Another limitation of this review is the exclusion of qualitative studies. This decision aligned with the review’s aims, which were to examine how routines and habits are currently measured and to identify the main outcomes assessed across studies. Therefore, the review focused on quantitative evidence to better map the volume and distribution of measurement approaches and outcomes. However, excluding qualitative studies may have limited a deeper understanding of how individuals with ADHD experience routines and habits in everyday life.

A further limitation is that many included studies relied on non-validated or study-specific measures. This made it more difficult to compare routine- and habit-related difficulties across studies, because similar behaviors were often described differently across questionnaires, even when they appeared to represent the same underlying construct.

A formal quality appraisal was not conducted because the purpose of this scoping review was to map the literature rather than evaluate methodological quality or compare the studies. As a result, the findings should be interpreted as a broad overview of the available evidence rather than as an assessment of the strength of that evidence.

The review was also limited by the heterogeneity of the included studies. Differences in ADHD presentation, comorbidities, age groups, medication status, and study methods may all have influenced the interpretation and generalizability of the findings. Although this heterogeneity reflects the complexity of ADHD, it also makes it difficult to draw conclusions that apply consistently across all individuals with the condition.

Finally, the included studies were conducted in more than 10 countries with different cultural norms related to sleep, diet, physical activity, and screen use. These contextual differences are important because the routines and habits described in each study may reflect local expectations and family practices rather than universal patterns.

## 5. Conclusions

Despite the limitations of this review, the findings suggest that individuals with ADHD often experience difficulties with routines and habits, especially in sleep, feeding, physical activity, and sedentary behaviors. These difficulties may affect daily functioning and participation. However, few interventions, programs, and assessment tools specifically address these areas. Clinicians, including occupational therapists and psychologists, can use these findings to screen for routine and habit-related difficulties as part of ADHD care and to support families or individuals in developing structured, realistic, and healthy routines and habits. For example, intervention planning may include strategies to improve bedtime routines, mealtime structure, physical activity patterns, and reduced sedentary time, rather than focusing only on ADHD symptoms. Therefore, future research and clinical practice need to consider routines and habits as essential aspects of ADHD care and as important outcomes for supporting daily functioning and participation.

## Figures and Tables

**Table 1:** Experimental Studies.

Author (Year)	Country	Study Type	Participant Characteristics	Intervention Description	Outcome Measures	Main Findings
Corkum et al. (2016)	Canada	RCT	22 ADHD, 39 TD (9.1 ± 1.98 years)	Better Nights/Better Days behavioral sleep program (parent manual + weekly telephone coaching) + actigraphy	CSHQ	Intervention group showed reduced sleep onset delay, bedtime resistance, and total sleep problems, with improvements maintained at 6-month follow-up; children with and without ADHD responded similarly.
Hiscock et al. (2015)	Australia	RCT	244 ADHD (5–12 years)	Sleep hygiene practices + standardized behavioral strategies (two consultation sessions + follow-up telephone call)	CSHQ; ADHD-RS	Behavioral sleep intervention reduced ADHD symptom severity and sleep problems, with effects sustained at 6 months and partially mediated by improved sleep.
Ola et al. (2021)	USA	Pilot feasibility and acceptability trial	31 ADHD (7.6 ± 1.4 years)	Lifestyle Enhancement for ADHD Program (LEAP): parent behavior management training + physical activity promotion + sleep and screen time strategies + Garmin activity tracking + private Facebook group	Feasibility and acceptability metrics (attendance, adherence, satisfaction); parent-reported routine and behavior questionnaires	LEAP was feasible and highly acceptable, with high parent adherence to sessions and activity tracking, and positive engagement with physical activity, sleep routines, and screen time management strategies.
Peppers et al. (2016)	USA	Pilot pre–post	23 ADHD (5–11 years)	Provider-instructed sleep hygiene education + prescriptive sleep routine (video instruction + individualized sleep plan)	CSHQ; Vanderbilt ADHD-RS (Parent Form)	Children showed significant improvement in sleep quality and reductions in ADHD symptoms following the sleep hygiene routine.
Shokravi et al. (2016)	Iran	RCT	56 ADHD (8.6 ± 1.57 years)	Sleep hygiene education (training session + booklet + two follow-up telephone calls + educational text messages)	CSHQ	Intervention group showed significant reductions in bedtime resistance, sleep onset delay, sleep duration, sleep anxiety, daytime sleepiness, and total CSHQ scores compared with controls.
Sonne et al. (2016)	United Kingdom	Within-subject baseline–intervention	13 ADHD (9.3 years)	MOBERO mobile routine support system (morning and bedtime routines + visual schedules + rewards)	CSHQ; ADHD-RS	Use of MOBERO was associated with reduced parent-rated ADHD symptoms, significant improvements in inattention and behavior, and improved sleep habits, with reductions in total CSHQ scores and most sleep subscales.

*Note.* RCT = randomized controlled trial; ADHD = attention deficit hyperactivity disorder; TD = typically developing; CSHQ = Children’s Sleep Habits Questionnaire; ADHD-RS = ADHD Rating Scale.

**Table 2:** Exploratory Studies.

Author (Year)	Country	Type of Study	Participants (N, Sex, Age)	Domain(s)
Becker et al. (2016)	USA	Cross-sectional	N = 147 (86 M/61 F) Mean age: 8.62 ± 1.17	Sleep
Björk et al. (2018)	Sweden	Cross-sectional	N = 48 (19 M/29 F) Mean age: 36 ± 11	PA, SB
Bussing et al. (2016)	USA	Mixed methods, cross-sectional	N = 148 (61 M/87 F) Mean age: 16.5 ± 1.30	Feeding
Eyuboglu and Eyuboglu (2018)	Turkey	Cross-sectional	N = 83 (64 M/19 F) Mean age: 8.8 ± 1.4	Sleep
García-Hermoso et al. (2024)	Spain	Ambispective cohort	N = 2609 (1014 M/1595 F) Mean age: 37.08 ± 1.51	PA
Golos et al. (2024)	Israel	Cross-sectional	N = 70 (55 M/15 F) Mean age: 5.18 ± 0.65	Sleep, SB
Gomes et al. (2023)	Portugal	Cross-sectional	N = 381 (194 M/187 F) Median age: 5	Sleep
Halt et al. (2023)	Finland	Cross-sectional	N = 130 (92 M/32 F/6 NR) Age: 16	Feeding, PA
Holton and Nigg (2020)	USA	Cross-sectional case–control	N = 184 (131 M/53 F) Mean age: 10.3 ± 1.5	Sleep, PA, SB
Hong et al. (2021)	Australia	Cross-sectional	N = 54 (30 M/13 F/11 NR) Mean age: 9.35 ± 1.11	Sleep, PA, SB
Islam et al. (2024)	Bangladesh	Cross-sectional	N = 542 (259 M/283 F) Mean age: 16.5 ± 1.3	Sleep, PA, SB
E. J. Kim et al. (2014)	South Korea	Cross-sectional	N = 12,350 (6010 M/6340 F) Mean age: 9.4 ± 1.7	Feeding
K. M. Kim et al. (2018)	South Korea	Cross-sectional	N = 16,831 (8352 M/8423 F) Mean age: 9.29 ± 1.71	Sleep
Lindstrom et al. (2020)	Georgia	Cross-sectional	N = 100 (40 M/60 F) Mean age: 20.8 ± 1.2	Study habits
Loewen et al. (2020)	Canada	Longitudinal cohort	N = 3436 (NR) Age: 10–11	Feeding, PA
Noble et al. (2011)	USA	Cross-sectional	N = 76 (47 M/20 F) Mean age: 7.6 ± 18 months	Sleep
Rodopman-Arman et al. (2011)	Turkey	Case–control	N = 44 (32 M/12 F) Mean age: 9.1 ± 1.5	Sleep
Rojo-Marticella et al. (2022)	Spain	Cross-sectional	N = 210 (151 M/59 F) Preschool: n = 42, mean age 5.18 ± 0.41/School-aged (n = 168, mean age 11.02 ± 0.71	Feeding
San Mauro Martin et al. (2021)	Spain	Cross-sectional	N = 57 (39 M/18 F) Age: 6–16	Feeding, SB
Sciberras et al. (2017)	Australia	Cross-sectional	N = 362 (271 M/90 F) Mean age: 9.5 ± 1.7	Sleep
Suchert et al. (2017)	Germany	Cross-sectional	N = 913 (492 M/421 F) Mean age: 15 ± 0.6	SB
Tong et al. (2016)	China	Cross-sectional	N = 785 (409 M/376 F) Age: 9–13	Feeding, SB
Wannapaschaiyong et al. (2024)	Thailand	Cross-sectional	N = 80 (60 M/20 F) Mean age: 5 ± 0.61	Sleep
Wu et al. (2016)	Canada	Longitudinal cohort	N = 4875 (2395 M/2480 F) Age: 10–11	Feeding, PA, SB
Zhang et al. (2024)	China	Cross-sectional	N = 120 (75 M/45 F) Mean age: 8.95 ± 1.07	Feeding, SB

*Note.* All ages reported in years unless otherwise indicated. NR = not reported; PA = physical activity; SB = sedentary behaviors.

**Table 3:** Outcome Measures.

Author (Year)	Outcome Measures
Becker et al. (2016)	CSHQ (sleep)
Björk et al. (2018)	Non-validated questionnaire (PA, SB)
Bussing et al. (2016)	Non-validated questionnaire (feeding)
Eyuboglu and Eyuboglu (2018)	CSHQ (sleep)
García-Hermoso et al. (2024)	MVPA (PA)
Golos et al. (2024)	FRI (sleep); YC-PEM (SB)
Gomes et al. (2023)	CSHQ (sleep)
Halt et al. (2023)	Non-validated questionnaire (feeding, PA)
Holton and Nigg (2020)	Lifestyle questionnaire (sleep, PA, SB)
Hong et al. (2021)	CSHQ (sleep); CLASS (PA); non-validated questionnaire (SB)
Islam et al. (2024)	SAS-SV (SB); non-validated questionnaire (sleep, PA)
E. J. Kim et al. (2014)	Non-validated questionnaire (feeding)
K. M. Kim et al. (2018)	Non-validated questionnaire (feeding)
Lindstrom et al. (2020)	Non-validated questionnaire (study habits)
Loewen et al. (2020)	PAQ-C (PA); FFQ (feeding)
Noble et al. (2011)	CSHQ (sleep)
Rodopman-Arman et al. (2011)	CSHQ (sleep)
Rojo-Marticella et al. (2022)	FFQ (feeding)
San Mauro Martin et al. (2021)	KIDMED (feeding); non-validated questionnaire (SB)
Sciberras et al. (2017)	CSHQ (sleep)
Suchert et al. (2017)	Non-validated questionnaire (SB)
Tong et al. (2016)	Non-validated questionnaire (feeding, SB)
Wannapaschaiyong et al. (2024)	CSHQ (sleep)
Wu et al. (2016)	Non-validated questionnaire (feeding, PA, SB)
Zhang et al. (2024)	Non-validated questionnaire (feeding, SB)

*Note.* CSHQ = Children’s Sleep Habits Questionnaire; PA = physical activity; MVPA = measures of moderate-to-vigorous physical activity (self-report); FRI = Family Routines Inventory; YC-PEM = Young Children’s Participation and Environment Measure; SB = sedentary behaviors; SAS-SV = Short Version of the Smartphone Addiction Scale; CLASS = Children’s Leisure Activities Study Survey-Parent Questionnaire; PAQ-C = Physical Activity Questionnaire for Children; FFQ = Food Frequency Questionnaire; KIDMED = Mediterranean Diet Quality Index for Children and Adolescents.

## Data Availability

All accessible data are contained in the manuscript.

## Supplementary Materials

The following supporting information can be downloaded at: https://www.mdpi.com/article/10.3390/bs16061000/s1, Table S1. Search Strategy.

## Abbreviations

The following abbreviations are used in this manuscript:

ADHD	Attention deficit hyperactivity disorder
MOHO	Model of Human Occupation
CSHQ	Children’s Sleep Habits Questionnaire
MVPA	Moderate-to-vigorous physical activity
SB	Sedentary behaviors
PA	Physical activity
FFQ	Food Frequency Questionnaire
KIDMED	Mediterranean Diet Quality Index for Children and Adolescents.
CLASS-PA	Children’s Leisure Activities Study Survey–Parent Questionnaire
PAQ-C	Physical Activity Questionnaire for Children
YC-PEM	Young Children’s Participation and Environment Measure
SAS-SV	Short Version of the Smartphone Addiction Scale
