# Supplementary material for: Daily Routines and Habits in Individuals with Attention Deficit Hyperactivity Disorder: A Scoping Review
Source: Behav Sci (Basel). 2026 Jun 15;16(6):1000. doi: 10.3390/bs16061000 (PMC13295911; doi:10.3390/bs16061000)
Supplement: Supplementary file 1 [file behavsci-16-01000-s001.zip › behavsci-4192260-supplementary.pdf]

Supplementary Materials

Table S1. Search Strategy

Database	Search term	Number of records identified
PubMed	("Attention Deficit Disorder with Hyperactivity"[Mesh] OR Attention-deficit-disorder* [tiab] OR Attention-deficit-hyperactivity-disorder* [tiab] OR hyperkinetic-syndrome* [tiab] OR minimal-brain-dysfunction [tiab] OR ADHD [tiab] OR ADDH [tiab])<br>AND<br>(routine* [tiab] OR habit* [tiab] OR lifestyle* OR life-style*[tiab])	1518
Scopus	TITLE-ABS-KEY ((attention-deficit-disorder* OR attention-deficit-hyperactivity-disorder* OR hyperkinetic-syndrome* OR minimal-brain-dysfunction OR adhd OR addh) AND (routine* OR habit* OR lifestyle* OR life-style*)) AND (LIMIT-TO (DOCTYPE , "ar") OR LIMIT-TO (DOCTYPE , "re"))	2889
CINAHL	(MH "Attention Deficit Hyperactivity Disorder" OR Attention-deficit-disorder* OR Attention-deficit-hyperactivity-disorder* OR hyperkinetic-syndrome* OR minimal-brain-dysfunction OR ADHD OR ADDH)<br>AND<br>(routine* OR habit* OR lifestyle*)	547
PsycInfo	(MAINSUBJECT.EXACT.EXPLODE("Attention Deficit Disorder") OR tiab(Attention-deficit-disorder* OR Attention-deficit-hyperactivity-disorder* OR hyperkinetic-syndrome* OR minimal-brain-dysfunction OR ADHD OR ADDH))<br>AND<br>(MAINSUBJECT.EXACT("Lifestyle") OR tiab(routine* OR habit* OR lifestyle* OR life-style*))	925
